# Dental education during the pandemic: Cross‐sectional evaluation of four different teaching concepts

**DOI:** 10.1002/jdd.12653

**Published:** 2021-05-27

**Authors:** Janosch Goob, Kurt Erdelt, Jan‐Frederik Güth, Anja Liebermann

**Affiliations:** ^1^ Department of Prosthetic Dentistry, University Hospital LMU Munich Munich Germany; ^2^ Department of Prosthetic Dentistry Center for Dentistry and Oral Medicine (Carolinum) Goethe‐University Frankfurt am Main Germany

**Keywords:** asynchronous, conventional lecture, livestream, pandemic, prerecorded PowerPoint, SARS‐CoV‐2, synchronous, Zoom conference

## Abstract

**Objectives:**

To evaluate the advantages of student satisfaction with and functionality of three digital teaching concepts during the SARS‐CoV‐2 pandemic compared to a conventional lecture setup.

**Methods:**

This cross‐sectional study was a survey‐based e‐learning research among dental students in the clinical study phase of a department of prosthetic dentistry at a German university hospital. A total of 44 questions were answered in four main sections: 1. general technical components; 2. acceptance; 3. evaluation and functionality; and 4. overall evaluation and grades of the various digital concepts. The use of Zoom conference, livestream, and prerecorded PowerPoint was compared to the conventional lecture setup (control group/CG). Data were analyzed using the Kolmogorov‐Smirnov test, followed by an exploratory data analysis and Cronbach's alpha test (*α* = 0.05).

**Results:**

Students were very satisfied with the provision, quality, and benefit of the digital concepts. The asynchronous concept was significantly more satisfying than the synchronous concepts in many aspects but was less successful in interactions. In the overall evaluation and grading, the asynchronous concept was rated significantly better than the other synchronous concepts (*p* ≤ 0.007), followed by Zoom conference, conventional lecture (CG), and livestream, while Zoom conference and the conventional lecture showed no significant difference (*p* = 0.784).

**Conclusions:**

Students significantly preferred the asynchronous concept to the synchronous concepts. The results suggested that asynchronous concepts are an effective and functional form of distance education during a pandemic. In general, digital teaching concepts are currently widely accepted for maintaining university education.

## 1 INTRODUCTION

The SARS‐CoV‐2 virus has spread all over the world and affected social life as well as all public institutions (such as universities).^1^, ^2^, ^3^, ^4^ Dental schools had to implement digital teaching concepts to offer web‐based interactions or digitalized contents to their students.^5^ Despite the availability of decent digital teaching concepts, the theoretical education of dentistry is mostly conventional through “face‐to‐face” lectures in an auditorium. The development of new digital teaching concepts in times of global pandemics, as well as the need for the digitalization of dental education, must be given priority, and improved in order to be better prepared. Previous studies have shown that online teaching with digital teaching concepts and the provision of digital media influences positively the interest in learning material and has a positive effect on the learning outcome of students.^6^ Students generally have an open attitude toward e‐learning courses.^7^ These investigations, however, do not reflect the current problems of the pandemic and were conducted at a time when the world was not affected by SARS‐CoV‐2. A recent study showed a wide acceptance of, and a generally positive perspective toward, the implementation of online teaching during the SARS‐CoV‐2 pandemic.^8^ However, anxiety, insecurity, a lack of social contacts, and depression currently influence the situation. A recent investigation has shown that there is a significant increase in psychological problems among students compared to the normal pre‐pandemic situation.^9^ It can be assumed that students agree to temporary purely digital teaching in order to flatten the curve of new infections, but the long‐term consequences of social distancing should not be underestimated. This is why universities must develop useful concepts to provide structured interactive digital teaching to guarantee students security in their education and learning success in order to maintain quality and avoid mental and psychological problems as far as possible.

The success of digital learning depends largely on the mindset and the interactive teaching styles of the specific university, as well as the students’ attitudes toward, and knowledge about, digital technologies.^10^ Digital teaching media can be divided into two categories: synchronous and asynchronous learning.^11^ Synchronous learning can be found, for example, in video chats such as Zoom conferences or livestream where lectures are transmitted in real time to the student's digital device. It is characterized as being more social through the ability to ask and answer questions in real time.^11^ In addition, synchronous communication helps e‐learners to feel more like participants than isolated individuals,^12^ which can be a mental advantage in times of social distancing. Asynchronous learning is an e‐learning concept where interactions between students and teachers take place independent of time and place^11^ and includes any recordings of teaching content that are not transmitted live to the students’ digital devices, such as prerecorded PowerPoint presentations with audio explanations, screencasts, podcasts, or videos. A key component of asynchronous e‐learning is flexibility.^11^ In fact, many people participate in asynchronous online courses because their flexible nature allows them to combine education with other daily commitments.^11^ The essential differences between synchronous and asynchronous learning are the communication via instant messages, direct feedback, and flexibility. The demand for universities is to provide a flexible, high‐quality digital teaching concept to keep dental education alive, not only for the current situation but also for a possible ongoing pandemic in 2021 and in the future.

This study aimed to evaluate the different specific advantages, the satisfaction, and the functionality of three different digital teaching concepts during the SARS‐CoV‐2 pandemic and compare them with the well‐known conventional lecture. With this knowledge it may be possible to create a pandemic teaching concept for universities with a view to helping them to be better prepared for future pandemics. The hypothesis tested states that there is no difference in students’ satisfaction and the functionality of the diverse digital teaching concepts evaluated in the current time of the SARS‐CoV‐2 pandemic and social distancing.

## 2 MATERIALS AND METHODS

During the pandemic period, the conventional lecture in the auditorium has been replaced by three digital teaching concepts. This cross‐sectional study was a survey‐based e‐learning research among 102 dental students in the clinical study phase (8th and 10th semester) of a department of prosthetic dentistry at a German university hospital.

**FIGURE 1:**
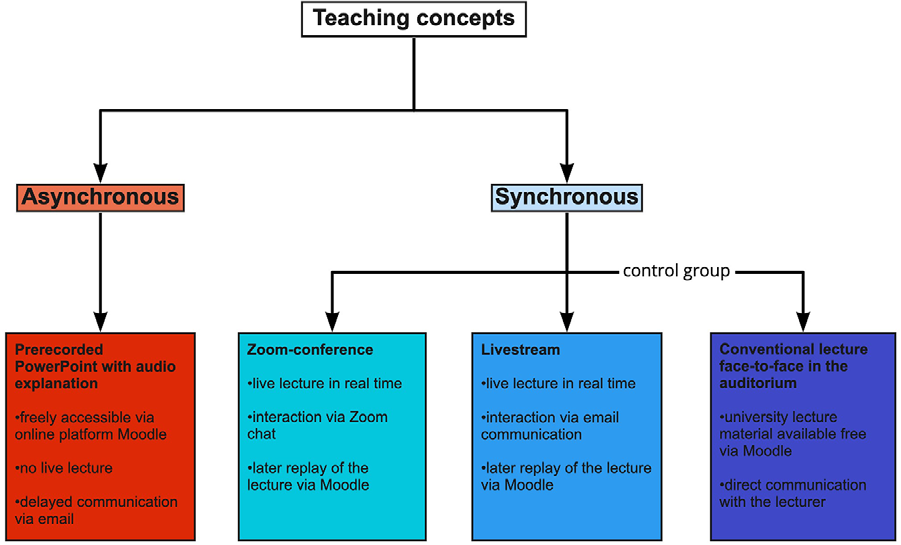
Overview of the teaching concepts evaluated

The series of lectures “Removable dental protheses I‐III” was presented through three different digital teaching concepts during the temporary closure of auditorium events in universities and compared with similar conventional lectures given the previous semester. All of the four teaching concepts (Figure 1) and lectures were presented by the same professor. The students were informed at the beginning of the semester about the digital course and the three different digital teaching concepts via email. Two of the three concepts were synchronous (Zoom conference and livestream), and the third was asynchronous (prerecorded PowerPoint presentations with audio explanations). Besides the free availability of the asynchronous prerecorded PowerPoint presentations, the other synchronous concepts were recorded and made available to the students for download afterwards. The following section explains the three digital teaching concepts:

1. Zoom conference: Zoom (Zoom Video Communications, Inc., San José, USA) is a communications technology that provides video telephony and can be used for distance education. The access link to the Zoom video conference was sent to the students via an already existing online learning platform (Moodle) of the university hospital. A Zoom conference enables the use of synchronous live video telephony conference for a variable number of students. The communication among the students is via the webcam of any computer, tablet computer, or smartphone. The teacher presented the lecture on a laptop with a webcam. The students followed the presentation slides and heard the teacher's voice as well as seeing the professor on a small screen. Questions were accepted via the Zoom conference integrated chat function and answered directly by the teacher via the microphone or a message. The students’ microphones were switched off to avoid an uncontrolled acoustic level.
2. Livestream via vMix: vMix (StudioCoast Pty Ltd, Robina, Australia) is a software that allows the creation of productions by adding multiple cameras, videos, images, and audio to a web stream. The software offers to display, record, and stream the lecture at the same time. Livestream enables a digital teaching format that is close to the conventional auditorium. Access to the online livestream was sent to the students via the platform (Moodle). The link led directly to the livestream, and the professor was filmed during his lecture in the auditorium of the university and transmitted live with a delay of about 50 s. Questions were accepted via email and answered via livestream or email by a separate moderator during the lecture.
3. Prerecorded PowerPoint presentation with audio explanation: The PowerPoint presentation was prerecorded and uploaded to the learning platform Moodle. The students could download the PowerPoint audio files with their access code and could watch the presentation offline. Questions about the presentation were answered via email communication.
4. Conventional lecture in the auditorium: To compare digital teaching with a conventional lecture, the conventional lecture from the previous semester with the same professor (before the lockdown) was chosen as the comparison lecture (control).

### 2.1 Evaluation

A total of 102 students were invited for an online evaluation. The questionnaire was designed by the authors and developed in cooperation with the Chair of Didactics. Participation in the survey was voluntary and there was no compensation for participation. All students were dental students in the clinical study phase (8th and 10th semester). The questionnaire was created using an online survey platform called Questionstar (Questionstar, Hannover, Germany) and consisted of 44 questions (Q), 37 of which were answered using a visual analog scale (VAS) and seven with fixed‐answer options (Table 1). The VAS ranged from 0% as “not true” to 100% as “entirely true.” The questionnaire was divided into the following four sections (S) with the headings: 1. general technical components; 2. general acceptance of the different digital teaching concepts; 3. evaluation of the three different digital concepts and the conventional lecture; and 4. Overall evaluation and grades. For better comparability of the different digital teaching concepts, the same questions were asked within the four concepts.

**TABLE 1:** Questionnaire with all questions and results used with median, IQR (interquartile range) and percentage of answers, separately

Question number	Question	Answer possibility	Median	IQR	Number (%)
1. General technical components					
1	At the beginning of the lecture, the teacher provided comprehensive information about the system requirements (e.g., hardware, software) for using the digital lecture.	VAS (visual analog scale) range: 0% as not at all true – 100% as entirely true	80.5	50.3	
2	The quality of the digital media and documents provided were very good.	VAS range: 0% as not at all true – 100% as entirely true	91.0	21.0	
3	The technical components of the used concepts worked trouble‐free.	VAS range: 0% as not at all true – 100% as entirely true	84.0	32.0	
4	Select the following options or briefly describe the problems if the technical component did not work:	I had a bad Internet connection. The system performance of my digital device is outdated, or my system did not work The format offered was technically not suitable Other			33 (61.1) 5 (9.3) 4 (7.4) 10 (18.5)
5	I felt disadvantaged because of missing or inadequate hardware for the digital lecture.	VAS range: 0% as not at all true – 100% as entirely true	0.0	17.0	
6	For the digital lecture I mainly used the following equipment:	Laptop/computer Smartphone Tablet computer Computer in a PC pool/CIP room at the university Computers in an institution outside the university (e.g., public library, Internet café) Other			82 (76.6) 6 (5.6) 19 (17.8) 0 (0.0) 0 (0.0) 0 (0.0)
7	For the digital lecture I mainly used the following network:	private WLAN mobile data public WLAN hotspot WLAN in a PC pool/CIP room at the university WLAN in an institution outside the university (e.g., public library, Internet café) Other			91 (85.0) 8 (7.5) 5 (4.7) 2 (1.9) 1 (0.9) 0 (0.0)
2. General acceptance of the various digital teaching concepts					
1	The digital course concepts support/promote my learning within the university.	VAS range: 0% as not at all true – 100% as entirely true	77.0	46.0	
2	The digital course concepts support/encourage my learning outside the classroom (at home).	VAS range: 0% as not at all true – 100% as entirely true	98.0	22.0	
3	The provision of digital courses increases my learning motivation.	VAS range: 0% as not at all true – 100% as entirely true	92.0	28.5	
4	I prefer the online lectures to the conventional/classical lectures at the university.	VAS range: 0% as not at all true – 100% as entirely true	82.5	46.8	
5	I can concentrate better in my private environment and am more receptive than in a conventional lecture at the university.	VAS range: 0% as not at all true – 100% as entirely true	87.0	36.0	
6	I appreciate my learning gain through the availability of digital courses.	VAS range: 0% as not at all true – 100% as entirely true	87.5	34.8	
7	Through the online availability of digital lectures (e.g., videos, prerecorded PowerPoint with audio explanation), I can deepen the teaching content (also later).	VAS range: 0% as not at all true – 100% as entirely true	100	8.75	
8	Thanks to the online availability of digital lectures, I am more flexible in my time management.	VAS range: 0% as not at all true – 100% as entirely true	100	0.0	
9	When using the online lecture, I miss the direct discussion with the teacher.	VAS range: 0% as not at all true – 100% as entirely true	31.5	57.5	
10	The teaching concepts and tools used in the digital course made the contact with the teacher (interaction/discussion with teacher) difficult.	VAS range: 0% as not at all true – 100% as entirely true	22.0	55.0	
11	In the future, I would like to see digital teaching concepts being used more widely.	VAS range: 0% as not at all true – 100% as entirely true	100	27.0	
3. Evaluation of the three different digital concepts and the conventional lecture					
Zoom conference					
1	The transmission quality of the media and documents of the Zoom online lecture was very good.	VAS range: 0% as not at all true – 100% as entirely true	89.0	28.0	
2	The technical component of the Zoom conference worked trouble‐free.	VAS range: 0% as not at all true – 100% as entirely true	87.5	30.3	
3	How do you estimate the potential of a Zoom conference in digital teaching for further dental education?	VAS range: 0% as not at all true – 100% as entirely true	84.0	38.0	
4	Additional helpful functions for the interaction with the teacher were provided. The use of the Zoom conference chat improved the interaction with the lecturer.	VAS range: 0% as not at all true – 100% as entirely true	82.5	40.0	
5	Overall, I am satisfied with the digital teaching format Zoom conference and its integration into teaching compared to my previous experience of a conventional lecture.	VAS range: 0% as very dissatisfied – 100% as very satisfied	86.0	38.8	
6	I have reviewed the online‐provided Zoom lecture again at a later date.	Yes No			82 (80.4) 20 (19.6)
Livestream					
1	The transmission quality of the media and documents of the livestream live lecture was very good.	VAS range: 0% as not at all true – 100% as entirely true	81.5	38.0	
2	The technical component of livestream worked trouble‐free.	VAS range: 0% as not at all true – 100% as entirely true	80.0	45.5	
3	How do you estimate the potential of livestream in digital teaching for further dental education?	VAS range: 0% as not at all true – 100% as entirely true	67.0	62.0	
4	Additional helpful functions for the interaction with the teacher were provided for me. The use of synchronous email communication with the teacher was sufficient.	VAS range: 0% as not at all true – 100% as entirely true	59.5	58.5	
5	Overall, I am satisfied with the Livestream digital teaching format and its integration into teaching, compared to my previous experience with classroom teaching.	VAS range: 0% as very dissatisfied – 100% as very satisfied	65.0	52.0	
6	I have reviewed the online‐provided livestream lecture at a later date.	Yes No			69 (67.6) 33 (32.4)
PowerPoint presentation with audio explanation					
1	The transmission quality of the media and documents of the prerecorded PowerPoint presentation with audio explanation was very good.	VAS range: 0% as not at all true – 100% as entirely true	100	15.0	
2	The technical component of the prerecorded PowerPoint presentation with audio explanation worked trouble‐free.	VAS range: 0% as not at all true – 100% as entirely true	100	13.0	
3	How do you estimate the potential of prerecorded PowerPoint presentation with audio explanation in digital teaching for further dental education?	VAS range: 0% as not at all true – 100% as entirely true	100	11.0	
4	Additional helpful functions for the interaction with the teacher (e.g., chat/email) were provided for me.	VAS range: 0% as not at all true – 100% as entirely true	74.5	41.3	
5	Overall, I am satisfied with the digital teaching format of prerecorded PowerPoint presentation with audio explanation and its integration into teaching compared to my previous experience in classroom teaching.	VAS range: 0% as very dissatisfied – 100% as very satisfied	98.0	21.5	
6	I have reviewed the online‐provided prerecorded PowerPoint presentation with audio explanation lecture at a later date.	Yes No			92 (90.2) 10 (9.8)
Conventional lecture					
1	The transmission quality of the media and documents of the conventional lecture in the auditorium was very good.	VAS range: 0% as not at all true – 100% as entirely true	85.0	33.0	
2	The technical component of the conventional lecture worked trouble‐free.	VAS range: 0% as not at all true – 100% as entirely true	84.0	36.0	
3	How do you estimate the potential of the conventional lecture for further dental education?	VAS range: 0% as not at all true – 100% as entirely true	77.0	49.0	
4	The interaction and discussion with the teacher worked well in conventional teaching.	VAS range: 0% as not at all true – 100% as entirely true	98.0	24.5	
4. Overall evaluation					
1	For what purposes do you use the available records of educational events? For self‐study For specific post‐processing of the learning contents As a substitute for attending teaching courses To prepare for the examination	VAS range: 0% as not at all true – 100% as entirely true	100 100 84.0 10.0	12.3 16.5 52.3 13.5	
2	What grade would you give the individual digital teaching concepts?	Very good (1) Good (2) Satisfactory (3) Sufficient (4) Defective (5) Insufficient (6)	Zoom: 2.0 Livestream: 2.0 PowerPoint: 1.0 Conventional lecture: 2.0	1.0 1.0 1.0 1.0	
3	How should teaching in dentistry be structured in the future in your opinion? Online teaching should replace conventional lectures Online teaching should complement conventional lectures Conventional lectures should dominate	VAS range: 0% as not at all true – 100% as entirely true	71.0 100 43.5	73.8 19.3 58.5	
4	What advantages do you see for yourself when using digital teaching concepts? I can follow the course on the go with my digital device. The courses available online enable me to acquire the teaching content more efficiently while saving time. I can integrate the recorded lecture flexibly into my everyday life.	VAS range: 0% as not at all true – 100% as entirely true	82.0 100 100	66.0 14.8 2.0	

The link for the survey was sent to the students via their private university email account for completion anonymously. The study was approved by the ethics committee of the Medical School (Project No. KB 20/030) and declared harmless. The answers to the VAS questions were entered by the students with a scroll bar on a line, which reflected the range from 0% to 100%. The seven questions with fixed‐answer options were answered with a click.

### 2.2 Data analysis

The questionnaires were analyzed with the statistical program SPSS 25 (IBM, New York, NY, USA) with a significance level of *p* = 0.05. The verification of normal distribution of the answers was carried out with the Kolmogorov‐Smirnov test, followed by an exploratory data analysis. The median values of the questions and the range of deviation of the interquartile range (IQR) were used for the mean values. The IQR describes the dispersion of the data ‒ exactly 50% of the data were within the IQR. The Kruskal‐Wallis test for independent samples was used to compare the results (S4). An additional statistical reliability analysis (Cronbach's alpha test) was carried out to secure the compilation of the questions and to test the internal consistency.

## 3 RESULTS

Of the 102 students (8th semester: 45 female, 13 male, average age 25.5 ± 3.3; 10th semester: 32 female, 12 male, average age 26.3 ± 2.6), all (100%) participated in the online survey with no dropout. None of the questions (0%) showed a normal distribution. Therefore, nonparametric tests with median values and IQR were applied. The results with median values and IQR are shown separately in Table 1.

### 3.1 General technical components (S1)

The median value for S1Q1 showed that the students were well prepared for the digital teaching concepts in a short time (median = 80.5%; IQR = 50.3). The students were satisfied with the quality of the digital media and the documents provided (S1Q2: median = 91.0%; IQR = 21.0). Working the technical components of the media used was mostly trouble‐free (S1Q3: median = 84.0; IQR = 32.0). Thirty‐two students described problems with the transmission of digital data caused by a weak Internet connection (S1Q4). Almost no‐one felt disadvantaged due to missing hardware for digital education (S1Q5: median = 0.0; IQR = 17.0). All students (100%) had a digital device with which to participate. To view and use the different digital teaching concepts, 76.6% of the students used a computer, 17.8% a tablet computer, and 5.6% a smartphone (S1Q6). To follow the digital teaching online, 85.0% of the interviewed students used their private Internet connection (Wi‐Fi) from home; only 7.5% used their mobile data, 4.7% used public Wi‐Fi, and 2.6% another Internet service provider (S1Q7).

### 3.2 General acceptance of the different digital teaching concepts (S2)

The provision of digital teaching courses not only encouraged learning within the university (S2Q1: median = 77.0%; IQR = 46.0) but above all also promoted learning outside the university (S2Q2: median = 98.0%; IQR = 22.0). The students’ agreement that the availability of digital courses increased their motivation toward the teaching content was based on a median of 92.0% with an IQR of 29.0 (S2Q3). With a median of 82.5% and an IQR of 46.8, students preferred digital teaching over conventional lectures (S2Q4); many students could concentrate better in their private environment than in the full auditorium (S2Q5: median = 87.0%; IQR = 36.0). In general, the progress of knowledge through digital teaching concepts was evaluated positively (S2Q6: median = 87.5%; IQR = 34.8). The lectures, which could be reviewed online at a later date, made it possible to deepen the learning content (S2Q7: median = 100%; IQR = 8.75), which also meant that the students became more flexible in their time management (S2Q8: median = 100%; IQR = 0). Missing the active discussion with the professor in the auditorium attracted widely dispersed views (S2Q9: median = 31.5%; IQR = 57.5). The students evaluated the tools for online discussion as sufficient (S2Q10: median = 22.0%; IQR = 55.0) and would like to see an increase in digital teaching concepts in the future (S2Q11: median = 100%; IQR = 27.0).

### 3.3 Evaluation of the three different digital concepts and the conventional lecture (S3)

In Section 3 the different teaching concepts were specifically compared and evaluated by the same questions.

**FIGURE 2:**
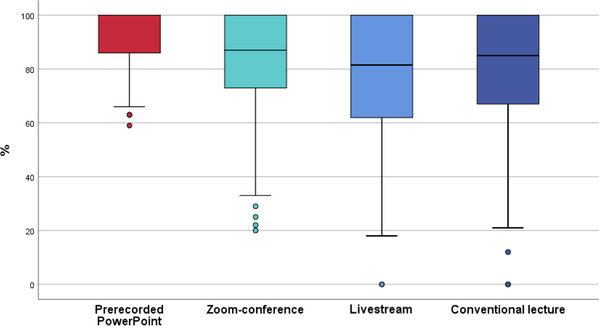
Boxplot of transmission quality of media and documents of all conventional/digital teaching concepts (%)

**FIGURE 3:**
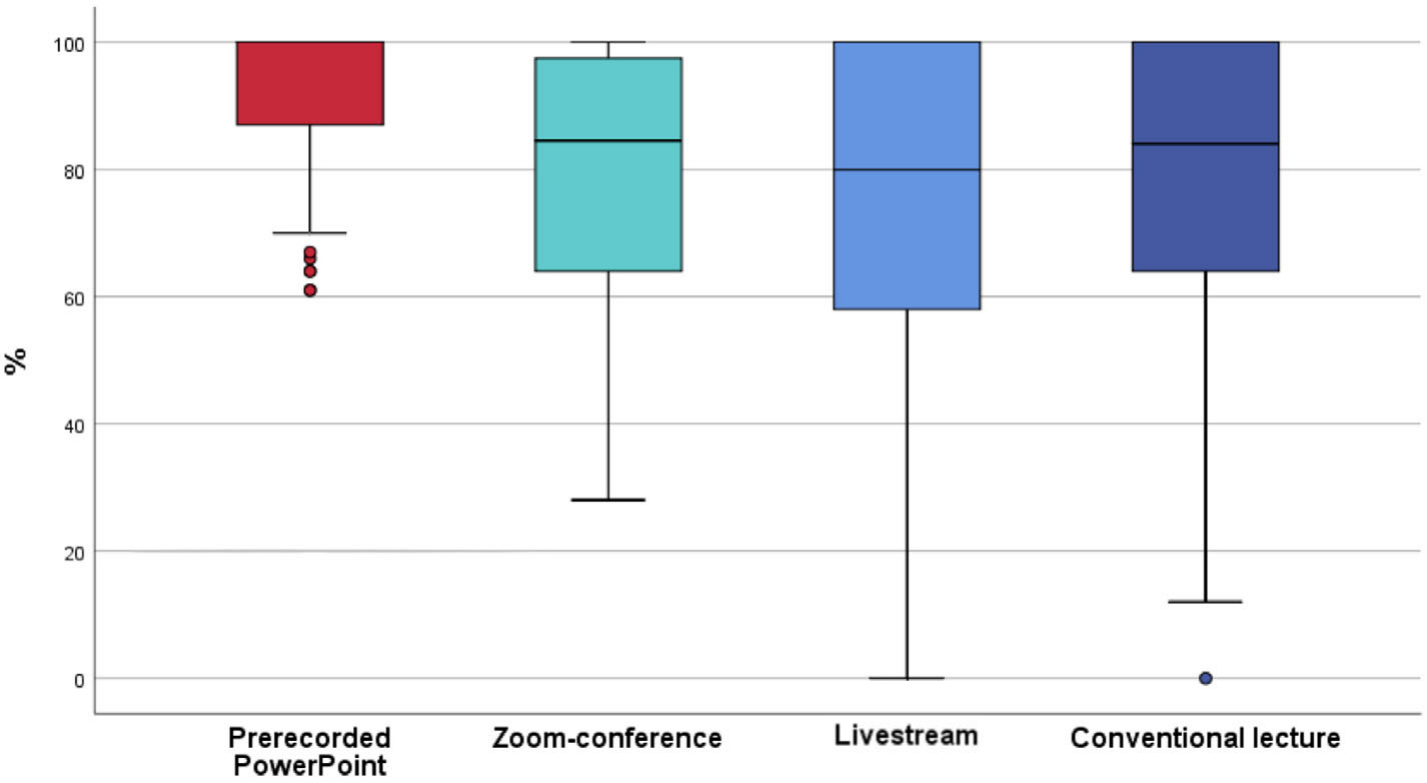
Boxplot of technical functionality of all conventional/digital teaching concepts (%)

In the first and second question (S3Q1+S3Q2), concerning the transmission quality (Figure 2) and technical functionality (Figure 3), the asynchronous PowerPoint presentation was rated significantly better than all other concepts (*p* < 0.001), with no significant differences (*p* > 0.999) between Zoom conference, livestream, and conventional lecture.

For S3Q3, in terms of the potential of the various teaching concepts for future education, the prerecorded PowerPoint presentation was seen as significantly different (*p* ≤ 0.002) from the other concepts. Zoom conference and conventional lecture (*p* = 0.618), and livestream, and conventional lecture, showed no significant differences (*p* = 0.356). Zoom conference and livestream were statistically significantly different (*p* = 0.002).

**FIGURE 4:**
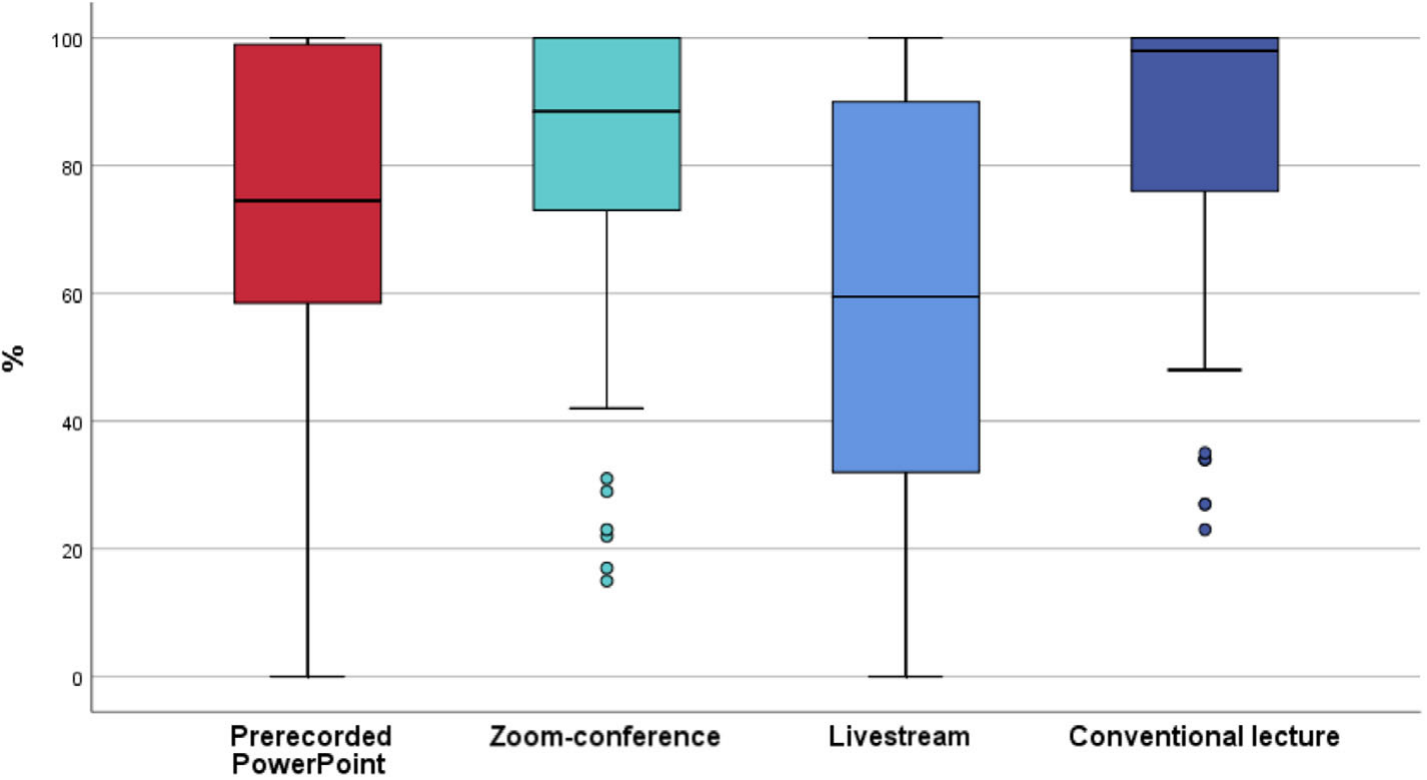
Boxplot of interaction and discussion between lecturer and student (%)

For S3Q4, in terms of interaction with the professor (Figure 4), Zoom conference and conventional lecture showed no significant difference (*p* > 0.999). Furthermore, Zoom conference and the prerecorded PowerPoint presentation presented no significant difference (*p* = 0.083).

In S3Q5, the conventional lecture was excluded from the statistics because it was already a part of university teaching. As regards the integration of digital teaching concepts in future teaching, there was no significant difference between the prerecorded PowerPoint presentation and Zoom conference (*p* = 0.265). The prerecorded PowerPoint presentation and livestream (*p* < 0.001) as well as Zoom conference and livestream (*p* = 0.001) differed significantly (*p* = 0.001).

The results of S3Q1‐S3Q5 for the direct comparison of the concepts are summarized in Table 2.

**TABLE 2:** Results of statistical analysis with median and IQR (interquartile range) of four teaching concepts for five different question sections

		Zoom conference	Livestream	Prerecorded PowerPoint	Conventional lecture
Q1	Median IQR	89.0^b^ 28.0	81.5^b^ 38.0	100.0^a^ 15.0	85.0^b^ 33.0
Q2	Median IQR	87.5^b^ 30.3	80.0^b^ 45.5	100.0^a^ 13.0	84.0^b^ 36.0
Q3	Median IQR	84.0^b^ 38.0	67.0^c^ 62.0	100.0^a^ 11.0	77.0^b,c^ 49.0
Q4	Median IQR	82.5^a,b^ 40.0	59.5^c^ 58.5	74.5^b^ 41.3	98.0^a^ 24.5
Q5	Median IQR	86.0^a^ 38.8	65.0^b^ 52.0	98.0^a^ 21.5	/

The letters ^a, b, c^ indicate statistically significant differences between the teaching concepts within the questions.

S3Q6 was evaluated descriptively. Out of 102 surveyed students, 82 reviewed online the Zoom lecture, 69 the livestream lecture, and 92 the prerecorded PowerPoint presentation.

### 3.4 Overall evaluation (S4)

Section 4 reports the results of the overall evaluation and the benefits of the various teaching concepts. With a high degree of consistency, the students used the available online teaching content for self‐study (median = 100.0; IQR = 12.3), for the specific follow‐up/debriefing of the learning content (median = 100.0; IQR = 16.5), and for the preparation for final examinations (median = 100.0; IQR = 13.5).

**FIGURE 5:**
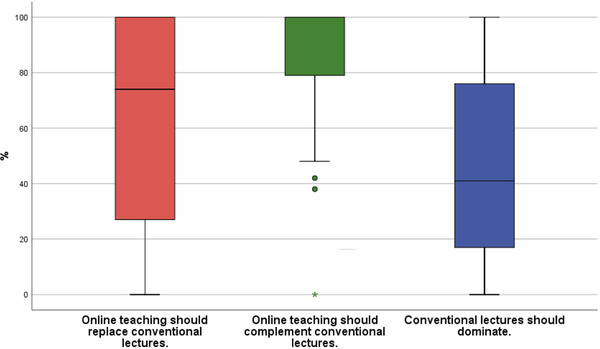
Boxplot of students’ vision of future teaching of dentistry without pandemic restrictions (%)

With regard to future teaching in dentistry without the restrictions of the pandemic, the combination of conventional lectures and digital teaching concepts had the highest acceptance (median = 100.0; IQR = 19.3). Purely digital teaching would also be accepted (median = 71.0; IQR = 73.8). Entirely conventional teaching, however, should not dominate the teaching in future (median = 43.5; IQR = 58.5) (Figure 5).

**Scoring**: In S4Q2, the students gave the digital teaching concepts grades from 1 to 6 (very good to insufficient; Table 3). The prerecorded PowerPoint presentation was significantly better rated than the other concepts (*p* ≤ 0.007). Zoom conference and conventional lecture showed no significant difference (*p* = 0.784) in the grade. The livestream was significantly different from the Zoom conference and prerecorded PowerPoint (*p* < 0.001) but not from the conventional lecture (*p* = 0.086).

**TABLE 3:** Results of statistical analysis with median and IQR (interquartile range) of four teaching concepts, separately

Group	Zoom conference	Livestream	Prerecorded PowerPoint	Conventional lecture
Median	1.71^b^	2.23^c^	1.32^a^	1.82^bc^
IQR	1.0	1.0	1.0	1.0

The letters ^a, b, c^ indicate statistically significant differences between the teaching concepts.

**Cronbach's alpha (α**) **test**: The compilation of the questions and the internal consistency of the questions in Section 4 were positive and could therefore be considered useful (Zoom conference: *α* = 0.852; livestream: *α* = 0.857; prerecorded PowerPoint: *α* = 0.799; conventional lecture: *α* = 0.906).

## 4 DISCUSSION

Due to the current situation of the SARS‐CoV‐2 pandemic, conventional lectures in dentistry had to be replaced by digital teaching concepts for theoretical content. The evaluation of the past semester under the SARS‐CoV‐2 restrictions supplies important and future‐oriented information for universities regarding the functionality of, and satisfaction with, digital concepts in regard to the ongoing pandemic and in the case of future pandemics. Universities and dental schools need to review and re‐evaluate this knowledge in order to guarantee professional and satisfactory education in the medical sector without quality loss or deficiencies in theoretical teaching. With this knowledge, not only can digital concepts be evaluated, but the data can also provide helpful information for the design of future teaching. Until the end of the year, teaching at universities in Germany will still take place online with digital concepts. Afterward, the situation and the infection rate must be reassessed before deciding whether a continuation of teaching at universities is justifiable. The current situation and the high infection rates suggest that theoretical teaching will continue to take place online in 2021.

The survey showed a high level of acceptance among the interviewed students in the clinical study phase, indicating that the implementation of digital teaching concepts is a current and highly important topic. In recent years, clinical dentistry has seen strong growth in computer technology and digital concepts,^13^ whereas theoretical teaching has remained more or less consistent in its conventional techniques. This was a big disadvantage at the beginning of the pandemic, as teachers and students had to familiarize themselves with a variety of existing digital teaching concepts. The hypothesis of the present investigation that there was no difference in the students’ satisfaction, and the functionality of the diverse digital teaching concepts was partially rejected due to the significantly better results of the asynchronous concept compared to the synchronous concepts.

In general, the survey confirms that all dentistry students who participated in the study own a digital device, have an Internet connection and were well prepared for the conversion from conventional to digital teaching under the circumstances of the pandemic. This means that the basic requirement for functioning digital teaching should be met. This contrasted with the fact that 32 of the 102 students complained that their Internet connectivity was too unstable to follow the live lectures in high quality and without interruption, which indicates that there are still deficits in the development, availability, and provision of fast Internet for students in the age of digitalization.

To the authors’ best knowledge, there are few data on the comparison of asynchronous and synchronous digital teaching concepts during pandemics. The present study can therefore only be compared to a certain extent with other investigations, as there has never before been a pandemic on this scale in the age of digitalization. In the present survey, there was a high level of acceptance of digital teaching courses in times of contact restrictions. The digital environment led to increasing motivation for the lecture topic of removable dental protheses, as almost all students attended the lecture, which was not compulsory. Similar results have been shown in other studies, such as a generally positive attitude toward digital dental courses^7^ and a high acceptance of e‐learning.^8^, ^14^ It has been described that digital distance teaching has a positive effect on the learning outcome and learning satisfaction among students.^6^ Earlier integration of digital teaching concepts would possibly further increase the acceptance of technology‐based digital teaching, since the implementation, and provision of digital media is nowadays generally regarded as positive. However, most studies concluded that a combination of conventional and digital teaching is the best approach to achieve the goals of educational success,^15^, ^16^, ^17^ had a consistently positive effect,^18^ and led to optimum satisfaction and performance.^19^ Currently, a combination of conventional and digital teaching is not possible due to government guidelines. Students would like to see an increased implementation of digital teaching concepts in the future, so in view of the current global pandemic situation and the diseases that may arise in the future, the knowledge about different digital teaching concepts and new VR (virtual reality) simulation technologies must be expanded.

The main objective of this study was to compare and evaluate the different digital teaching concepts with each other and compare them with the conventional lecture in terms of functionality, acceptance and satisfaction, in order to develop a professional teaching concept.

Despite the spontaneous change from conventional to purely digital teaching, there were generally no major problems. Theoretical teaching could be kept consistent, which was reflected in the results of the present study. In terms of transmission quality, the prerecorded PowerPoint presentation presented better results than the other teaching concepts (S3Q1). This may be due to the simplicity of the asynchronous format; after downloading the file, the lecture is available in the highest quality on the students’ digital devices, which enables a geographic and temporal flexibility of place and time^16^, ^20^ and allows education to be combined with other daily commitments.^11^ The technical components of the various digital concepts mostly worked without problems (S3Q2). The significantly better performance of the asynchronous format may be due to the fact that synchronous concepts are technically more sensitive.^20^ Minor software issues could be solved quickly in individual cases. In the case of further implementation of digital teaching concepts and the digitalization of theoretical dental education, students consider the prerecorded PowerPoint presentation to be the best and easiest way to transmit theoretical dental knowledge in the case of contact restrictions. Nevertheless, the direct discussion between professor and students should not be ignored in synchronous or asynchronous teaching and must therefore be critically assessed. Previous studies have underlined the significance of interaction in education and have stated that learning is highly influenced by interaction, collaboration, and social exchange.^21^, ^22^ Moreover, the interactions between lecturer and students could positively influence the motivation, attitude, success, and satisfaction of students.^23^, ^24^, ^25^, ^26^ In the present survey, students were given the opportunity to ask the professor questions and lead a discussion via email or a live chat function. As expected and as already shown in other studies,^20^ the communication and discussion was rated best in the conventional lecture, but the synchronous Zoom conference live chat function was also considered an uncomplicated and good way of providing high‐quality interaction. The synchronous communication and discussion was described as beneficial and more social, as students felt more like part of an active discussion, with increasd mental excitement, and motivation.^11^ Interviews showed that many e‐learners perceived synchronous communication as “more like talking” compared to asynchronous communication.^11^

One of the main reasons for the wide acceptance of digital teaching is that the content can be viewed again at a time and place of one's choosing,^20^, ^24^ which improves follow‐ups and examination preparation. This statement was confirmed in the present investigation by the large number of later views on the 24/7 online platform “Moodle.”

In a direct comparison of the concepts by school grades from 1 to 6 (very good to insufficient), the PowerPoint presentation was rated significantly best, followed in the ranking by Zoom conference, conventional lecture, and livestream (Table 3). However, the study perhaps does suggest that a certain level of asynchronous consumption of content is favored over live consumption. Also, when it comes to discussion of content during/after consumption, it appears that a hybrid of live chat and asynchronous email is preferred. The result indicated by the students is clear with regard to the design of future teaching: the combination of conventional lecture and digital teaching had the highest acceptance (Figure 5).

On the one hand, a strength of the present survey is that all four teaching concepts were presented by the same professor over a short period, thus minimizing the influence of different personalities on the evaluation. The design, motivation, and interaction of the professor can influence students’ satisfaction and acceptance. On the other hand, some limitations of the present investigation should be mentioned. Both synchronous formats offer asynchronous replay in the post‐processing, which the majority of respondents took advantage of, whereas the asynchronous lectures were not digitally captured, therefore adding the limitation of memory. Additionally, for the control group it was assumed that students had to rely on their memory/experience of attending a conventional pre‐pandemic lecture. These memories may be biased by the time, and this was therefore assessed as a limitation. The survey took place at one single institution, so the results may be difficult to generalize. Furthermore, the influence of one professor on the result cannot be assessed. Poor teaching by the professor would probably bias the results negatively, so it would be interesting if the results were similar in a multi‐center study at different dental universities with different professors. The theoretical outcome of the different concepts was not evaluated either, leading to subjective outcomes. In addition, there is significantly increased mental stress among university students during the pandemic compared to normal education.^9^ In the present investigation, possible psychological and mental influences in terms of satisfaction were not examined and could be a basis for further psychological surveys.

## 5 CONCLUSION

This study found that theoretical dental education through digital teaching has worked satisfactorily in times of a global pandemic. Despite the spontaneous switch from conventional to digital education, students were very satisfied with the teaching provided by the various digital teaching concepts. The results reflected the teaching situation during a global pandemic and can only be compared to a certain extent with other studies considering digital education. Within the limitations of the investigation, this study found that students significantly preferred the asynchronous PowerPoint presentation to synchronous concepts in many aspects. This may be due to the simplicity of the asynchronous format: flexibility in time and place and easy handling without technical problems. The result perhaps does suggest that asynchronous consumption of content is favored over live consumption and offers effective distance education in times of pandemics. Universities should remain focused on developing and improving synchronous and asynchronous concepts, especially in the area of interaction, as a significant part of teaching thrives through direct faculty/student and student/student interactions. Additional research is needed to compare lecture formats of different universities for the preclinical and clinical didactic teaching of dental students.

## CONFLICT OF INTEREST

The authors declare that there is no conflict of interest that could be perceived as prejudicing the impartiality of the research reported.
